# High-Temperature Performance Evaluation of Asphaltenes-Modified Asphalt Binders

**DOI:** 10.3390/molecules25153326

**Published:** 2020-07-22

**Authors:** Amirhossein Ghasemirad, Nura Bala, Leila Hashemian

**Affiliations:** Department of Civil and Environmental Engineering, University of Alberta, Edmonton, AB T6G 1H9, Canada; ghasemir@ualberta.ca (A.G.); bala1@ualberta.ca (N.B.)

**Keywords:** modified asphalt binder, asphaltenes, SARA, high-temperature performance, rheology, chemical composition

## Abstract

Asphalt binder comprises four main fractions—asphaltenes (A), saturates (S), aromatics (A), and resins (R)—referred to as “SARA”. Asphaltenes plays an important role in determining the linear viscoelastic behavior of asphalt binders. In this research, asphaltenes are added as a distinct modifier to improve the performance properties of asphalt binder. The modified binders are aged using a rolling thin film oven. A dynamic shear rheometer is then used to measure the rheological properties of the binders at high temperatures. Changes in the chemical composition of the modified binders are also studied through the determination of SARA fractions, using precipitation and gravity-driven chromatography methods. The rheological results show that asphaltenes improve the stiffness and elasticity of asphalt binder. It is also shown that the addition of asphaltenes raises the high Performance grade (PG) temperature of the asphalt binder, with every 6% of asphaltenes added resulting in a one-interval increase in high PG temperature grade. SARA analysis shows that the increase in polar fraction content due to the addition of asphaltenes causes the stiffness, elasticity, and viscosity of asphalt binders to increase. The results indicate that asphaltenes are an effective yet inexpensive additive to improve asphalt binder properties at high temperatures.

## 1. Introduction

Asphalt pavements are the most common type of pavement in North America, making them an important infrastructure asset. Asphalt mixture is composed of asphalt binder, aggregates, and fillers. In some cases, additives or modifiers are also added to improve the mixture properties [1,2]. Asphalt binder is a hydrocarbon product produced in crude oil refineries, mainly through fractional distillation [3]. After separation of lighter fractions (liquid petroleum gas, gasoline, aviation fuel, kerosene, etc.), the heaviest fraction taken from crude oil distillation, which is a complex mixture of high molecular weight hydrocarbons, is processed further to obtain asphalt binder [3]. Although in the pavement industry, asphalt binder is used as a binding agent for the aggregates, it also adds several characteristics to the asphalt mixture and plays an important role in determining distresses during the pavement’s service life [4]. Therefore, it is important to ensure that the asphalt binder used in the production of asphalt mixture performs well under different environmental conditions.

Increasing traffic demand, harsh weather conditions, and the tendency for infrastructure operators to reduce the cost (and, therefore, the frequency) of maintenance are among the major reasons for asphalt performance improvement strategies [3]. This has motivated researchers to seek ways to modify conventional asphalt binders to improve their performance and extend their service life [1,5]. In this regard, polymers, which are one of the most common materials used in asphalt modification, have significantly enhanced asphalt mixtures in terms of performance [6]. Despite the improvements recorded with these modifiers, some drawbacks are also associated with the use of polymers for asphalt binder modification. Perhaps the most notable disadvantage is polymer-modified asphalt being much more expensive than conventional asphalt binder [7,8]. Furthermore, phase separation may occur during the storage and application of polymer-modified asphalt binders [9,10,11].

Recently, due to increasing public concerns about sustainability, and given the high cost of some modifiers, the application of waste materials to improve asphalt binder properties has gained more attention by researchers [12,13]. Asphaltenes are one of the waste materials obtained through the deasphaltation process in oil sand asphalt binder production. Despite their high rate of production, asphaltenes have generally been considered a waste, with minimal value and few useful applications in industry [14,15]. However, there have been some efforts recently to make use of asphaltenes, including the development of a gasification technique to convert asphaltenes to gas fuel [16]; however, gasification is an expensive process that generates a considerable amount of environmental pollution [15].

According to its polarity, asphalt binders can be classified by chemical composition into saturates, aromatics, resins, and asphaltenes—collectively referred to as “SARA” [17,18]. The polarity of asphalt binder compounds and their interactions, in turn, play important roles in influencing the rheological properties of the asphalt binder [19].

The polar fractions of asphalt binder—asphaltenes and resins—account for its elastic behavior, while the non-polar fractions—saturates and aromatics—are responsible for its viscous behavior [20,21]. Due to differences in polarity, asphaltenes particles may agglomerate and become unstable in the surrounding matrix of the remaining fractions [22,23].

Two general methods of altering asphaltenes content in asphalt binders have been identified in the literature. The first is to use a mechanical stirrer to mix the asphalt binder with materials rich in asphaltenes [24,25], while the second is to add the asphaltenes separated from the asphalt binder, by n-heptane extraction, back into the binder [20,26]. The second method has the advantage of the asphaltenes content being adjustable, which has made this procedure more popular and has led to the emergence of the concept of artificial bitumen [27].

Previous investigations have shown that the temperature susceptibility of an asphalt binder decreases with an increase in asphaltenes content [8,25]. On the other hand, the addition of asphaltenes could improve the resistance of asphalt binders against aging [8]. Furthermore, it has been shown that it is the asphaltenes content that is primarily responsible for the elastic component of an asphalt binder’s viscoelastic response [20,26,28]. Higher asphaltenes content causes the asphalt stiffness to increase, which in return decreases the penetration and creep compliance [20,24], and increases the binder’s viscosity [18]. The abrupt change in asphalt binder properties resulting from the addition of asphaltenes is indicative of a fortifying of the network formed by the polar fractions (mainly asphaltenes) within the asphalt binder; a phenomenon which gives elastic characteristics to the binder [26,28].

Based on a review of the existing literature, to the authors’ knowledge there has been no investigation of the effects of using asphaltenes produced as a waste material in crude oil refineries as a distinct modifier in performance improvement of asphalt binders. The present study thus aims to investigate the high-temperature performance of asphalt binders modified with different levels of asphaltenes content in specific reference to binders produced at oil refineries.

The main objective of this study is to investigate the rheological performance of asphalt binders modified with asphaltenes derived from Alberta oil refineries at high temperatures, as well as their chemical compositions.

## 2. Materials and Test Methods

### 2.1. Materials

#### 2.1.1. Asphalt Binder

The asphalt binder used for this study was a straight-run asphalt binder with a performance grade (PG) of 70–22, and Table 1 shows the specifications of this binder

#### 2.1.2. Asphaltenes

Asphaltenes are a macro-polar structure that is found in solution within the oil matrix, and there are a few methods to extract asphaltenes from their sources, among which solvent dissolution, or solvent deasphalting, is the one that commonly used [22,38]. The solvent deasphalting process consists mainly of precipitation of asphaltenes by using n-alkanes as an anti-solvent [39]. The asphaltenes used in this research were produced in northern Alberta facilities from SAGD produced Athabasca bitumen, by adding a non-polar solvent (e.g., typically NC3 to NC7) in enough quantity to the oil matrix, in order to disrupt the solubility of the asphaltenes and force them to precipitate.

**Figure 1:**
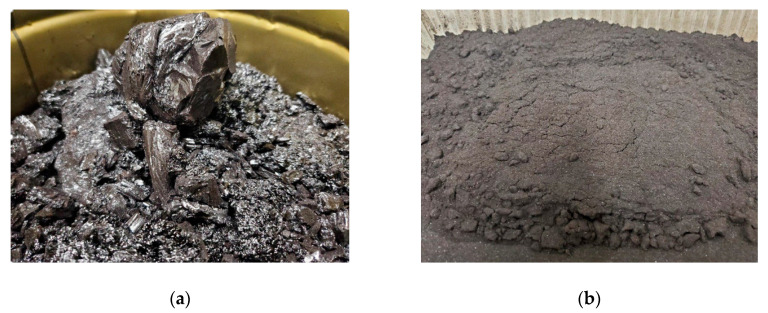
Asphaltenes: (**a**) solid form; (**b**) powder form after passing through a #100 sieve.

Asphaltenes have been considered to be a waste, with minimal value and few potential applications in industry. The asphaltenes used in this study were obtained in chunk form, as shown in Figure 1a. In order to make the mixing process more effective and provide adequate surface area for mixing, the solid asphaltenes were ground into powder form before adding them to asphalt binder, as shown in Figure 1b. Asphaltenes particles passing through a #100 sieve with an opening size of 150 microns were used to modify the asphalt binder.

### 2.2. Test Methods

#### 2.2.1. Preparation of Asphaltenes-Modified Asphalt Binders

All modified binders were prepared using a high shear mixer (L5M-A model, Silverson Co., East Longmeadow, MA, USA). In order to prevent aging at elevated temperatures during mixing, a hot plate was used to maintain the temperature of the asphalt binder at 140 ± 5 °C while mixing. The desirable amount of asphaltenes was then added to the heated asphalt binder and mixed at a rotation speed of 2000 rpm for 60 min in order to achieve a uniform blend.

In order to investigate the effects of asphaltenes across a wide range of asphaltenes content levels, asphaltenes-modified binders were prepared by adding 0% to 20% asphaltenes content (as a proportion of the weight of the asphalt binder) at increments of 3%, with the exception of the last mix, for which the asphaltenes content was increased from 18% to 20%.

#### 2.2.2. Aging Process and Aging Indices

In order to simulate the short-term aging that occurs in the asphalt binder during mixing in asphalt plants, a rolling thin film oven (RTFO) was used to age the asphalt binders in accordance with the AASHTO T240 standard [40]. The impact of asphaltenes on the aging characteristics of asphalt binders was studied by estimating the aging indices. These indices, are defined as the ratio of a performance parameter of the aged binder to that of the unaged binder [41,42]. Two aging indices, complex modulus aging index (CAI) and phase angle aging index (PAI), were estimated in this study using Equations (1) and (2), respectively [41]:

CAI = (aged complex modulus)/(unaged complex modulus),(1)

PAI = (aged phase angle)/(unaged phase angle),(2)

where a higher value aging index indicates a more severe impact of aging on the properties of the asphalt binder [41,43].

#### 2.2.3. Dynamic Shear Rheometry (DSR)

To characterize the viscoelastic behavior of asphalt binders at high temperatures, samples were tested according to the AASHTO T315 standard [44] using a dynamic shear rheometer (DSR) (Smartpave 102 model by Anton Paar, Co., Ltd., Graz, Austria). DSR applies sinusoidal shear loadings to samples and measures the stress and resulting strain response. Using these data, this device is able to calculate the complex shear modulus (G^*^), and phase angle (δ) of viscoelastic materials, where G^*^ is the ratio of maximum applied shear stress to the maximum value of strain, and δ is the time lag between shear stress and shear strain response. The complex shear modulus, in turn, is representative of the material’s stiffness, while the phase angle is an indicator of how elastic or viscous the material behaves. According to AASHTO M320 standard [45], at a loading frequency of 1.59 Hz, the temperatures at which the parameter G^*^/sinδ drops below 1.0 kPa and 2.2 kPa, would be recorded as the binder’s failure temperatures in regard to rutting resistance for unaged and RTFO-aged binders, respectively. The lower of these two temperatures is thus identified as the high PG grade of the binder.

#### 2.2.4. Rotational Viscosity

In this study, a Brookfield rotational viscometer was used to measure the viscosity of the asphalt binders at elevated temperatures in accordance with AASHTO T316 standard [46]. In order to ensure adequate workability of the asphalt mixture, AASHTO M320 standard [45], which restricts the maximum viscosity of binders at 135 °C to 3 Pa·s, was applied. The viscosities of the binders were investigated across a wide range of high temperatures (135 °C to 185 °C) in order to calculate the mixing and compaction temperature ranges of the asphalt binders. According to Superpave, compaction specimens require mixing and compaction under equiviscous temperature conditions corresponding to 170 ± 20 mPa·s and 280 ± 30 mPa·s, respectively.

#### 2.2.5. SARA Analysis

SARA analysis was conducted to separate different fractions of asphalt binders. The SARA test is composed of two main techniques: solvent dissolution to separate asphaltenes from maltenes, and gravity-driven chromatography to separate different fractions of maltenes. In this process, asphaltenes are first separated from the sample according to ASTM D6560 standard [47] using n-heptane as the solvent. Saturates, aromatics, and resins fractions are then determined using the clay-gel adsorption chromatography method in accordance with ASTM D2007 standard [32].

In order to quantify the stability of the asphaltenes in the maltenes phase, the colloidal index (CI) was calculated for the asphalt binders using Equation (3) [48,49].

CI = (Asphaltenes in wt% + Saturates in wt%)/(Resins in wt% + Aromatics in wt%),(3)

where a lower value of CI represents a higher stability of asphaltenes micelles in the asphalt binder. In this regard, according to Lesueur [18], for CI values greater than 1.2, the asphaltenes fraction tends to be unstable within the maltenes matrix. When the CI value is between 0.7 and 1.2, the asphaltenes stability is uncertain, while, for CI indices less than 0.7, the asphaltenes fraction is stable.

## 3. Results and Discussion

The results and analysis from different tests are available in this section. All the results presented in different tables and figures are the average of at least three repetitions, with the maximum allowable standard deviation of 10%.

### 3.1. PG Grading Results

In order to determine the high PG grade of the binders, DSR testing was conducted on all unaged and RTFOT-aged control and asphaltenes-modified binders. The results for high PG grading of asphalt binders are presented in Table 2. It can be seen that a noticeable increase in the high PG grade of asphaltenes-modified binders was observed, as compared to the base binder; this increase indicates a considerable improvement in resistance against permanent deformation. It can be concluded from Table 2 that, on average, a one-interval increase in high PG of the asphalt binder can be expected as a result of addition of every 6% asphaltenes. This enhancement in high PG grade is comparable with the improvement associated with some other well-known additives such as crumb rubber [11].

**Figure 2:**
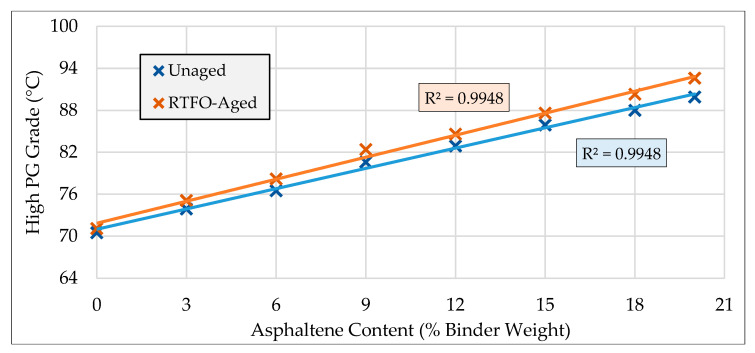
PG grading results for unaged and RTFO-aged binders.

The PG grade improvement of the binder at high temperatures corresponding to the addition of asphaltenes for both unaged and rolling thin film oven (RTFO)-aged binders is represented graphically in Figure 2. As can be seen in the figure, at both states of aging, the rate of improvement of the asphalt binder’s performance at high temperatures is roughly proportional to the increase in asphaltenes content. On the other hand, the slope of the RTFO-aged plot is more inclined than that of the unaged one, suggesting that the contribution of asphaltenes to rutting resistance increases as the binder ages.

### 3.2. Rheological Properties

**Figure 3:**
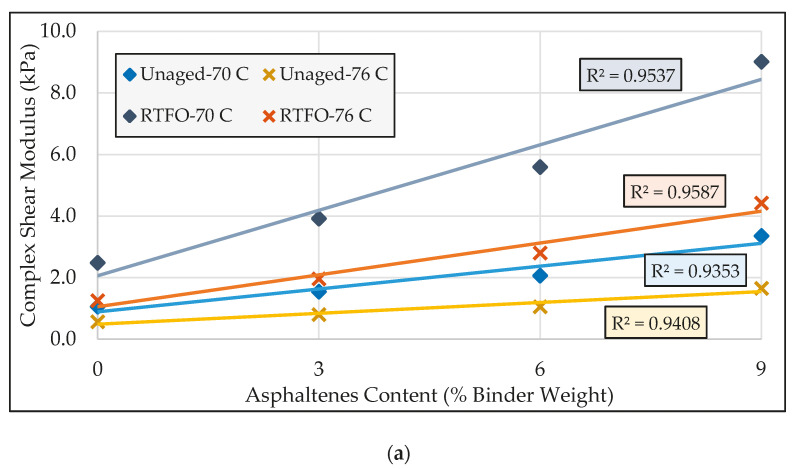

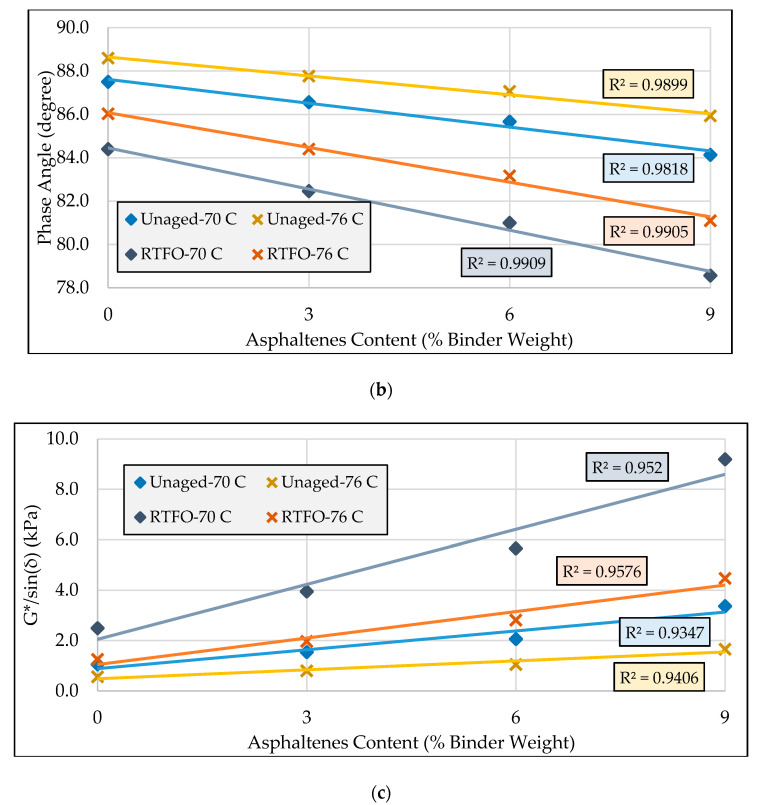
Comparison of rheological properties of the first group of modified binders: (**a**) complex shear modulus; (**b**) phase angle; (**c**) rutting factor.

**Figure 4:**
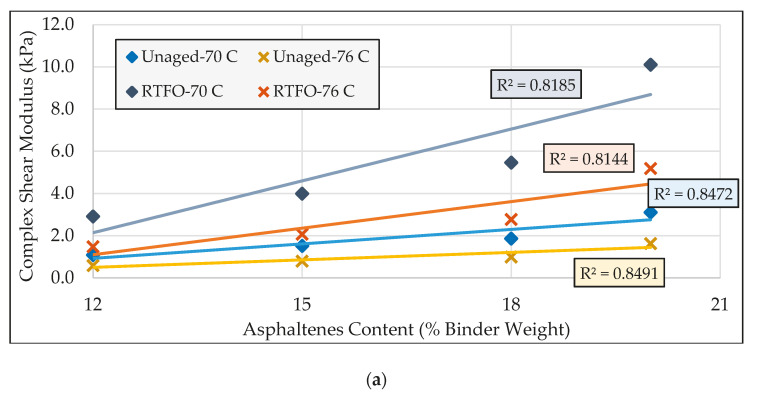

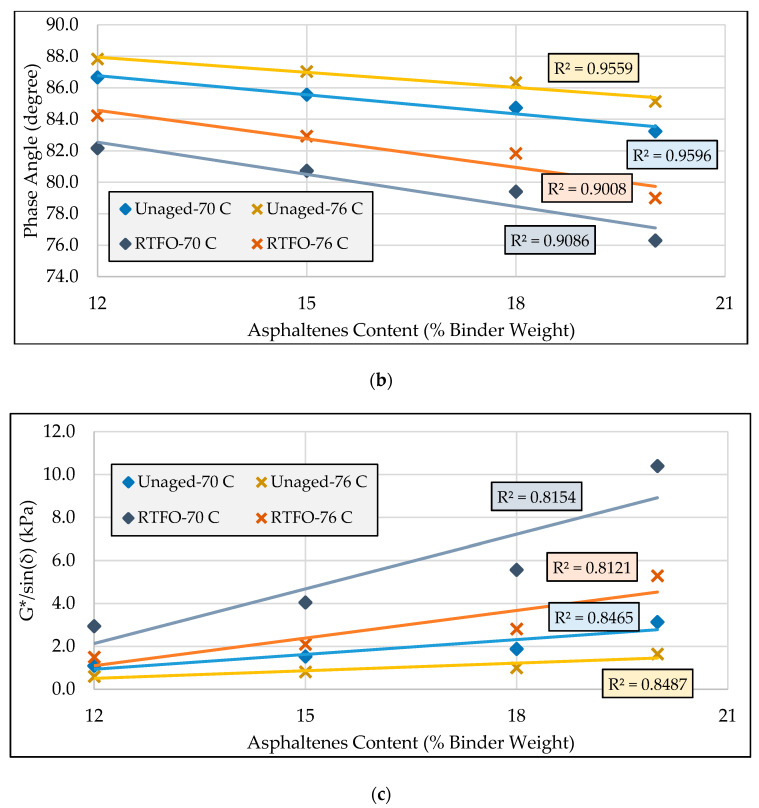
Comparison of rheological properties of the second group of modified binders: (**a**) complex shear modulus; (**b**) phase angle; (**c**) rutting factor.

The modified binders were divided into two groups: the first group included the binders containing 0%, 3%, 6% and 9% asphaltenes content, and the second category consisted of asphalt binders modified with 12%, 15%, 18%, and 20% asphaltenes content (relative to the binder weight). Figure 3 and Figure 4 compared the rheological specifications of the asphalt binders in the first and second groups, respectively.

From Figure 3 and Figure 4, it can be seen that increasing asphaltenes content was found to noticeably increase the complex shear modulus and decrease the phase angle, indicating an improvement in stiffness and elasticity of the asphalt binder at high temperatures. According to Figure 3a and Figure 4a, the G^*^ plots are steeper in the RTFO-aged state than in the unaged state. Furthermore, this higher rate of improvement was found to be more significant at higher temperatures. In the case of phase angle (δ), in reference to Figure 3b and Figure 4b, the rate of increase in elasticity in the aged state was found to be noticeably more than that in the unaged state. These observations reveal that the impact of asphaltenes on improving high-temperature performance parameters is more pronounced as the binder ages.

According to Figure 3a,b and Figure 4a,b, at relatively lower temperatures, the differences between RTFO-aged rheological parameters and corresponding unaged parameters were found to be higher. From this observation it can be inferred that, as the temperature decreases, asphaltenes show a more significant impact in terms of increasing the asphalt binder’s stiffness and elasticity due to aging. Given that higher values of stiffness and elasticity adversely affect the cracking resistance at relatively lower temperatures, it can be expected that the possibility of cracking would increase at lower temperatures with increased asphaltenes content.

As shown in Figure 3 and Figure 4, under the same aging condition, the rate of modulus change was found to increase as the temperature decreases, which implies that asphaltenes have more impact on the shear modulus at lower temperatures. The relatively consistent slope in phase angle plots, however, reflects the similar impact of asphaltenes on this parameter at lower and higher temperatures.

The same trends as those observed in the complex modulus plots can be seen in the rutting factor (G^*^/sin δ) charts shown in Figure 3c and Figure 4c. These trends serve to confirm that, as the binder ages, the impact of asphaltenes on improving rutting resistance becomes more effective. Furthermore, the rutting resistance resulting from the addition of asphaltenes increases at a higher rate as the temperature decreases.

### 3.3. Aging Indices

**Figure 5:**
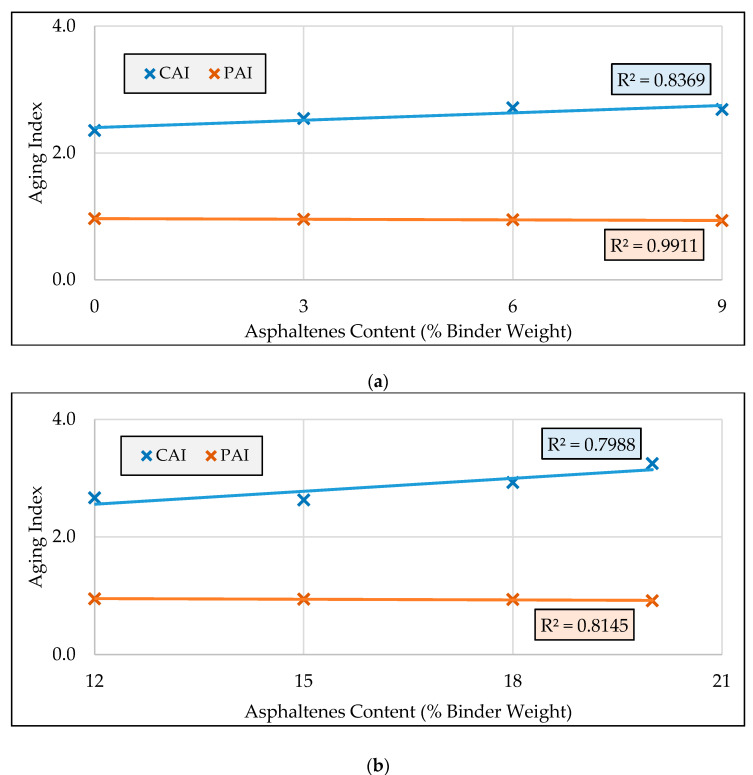
Aging indices for asphaltenes-modified binders: (**a**) aging indices of the first group of modified binders at 70 °C; (**b**) aging indices of the second group of modified binders at 82 °C.

The aging indices for the two groups of modified binders (at 70 °C and 82 °C) are presented in Figure 5a,b, respectively. According to these figures the phase angle aging index values for all modified binders remained consistently around 1.0 during aging, indicating that regardless of asphaltenes content aging has almost no effect on the phase angle and elasticity of the binder. However, in the case of the complex modulus aging index, generally, the addition of asphaltenes was found to increase the CAI for both groups of binders.

### 3.4. High-Temperature Viscosity

**Figure 6:**
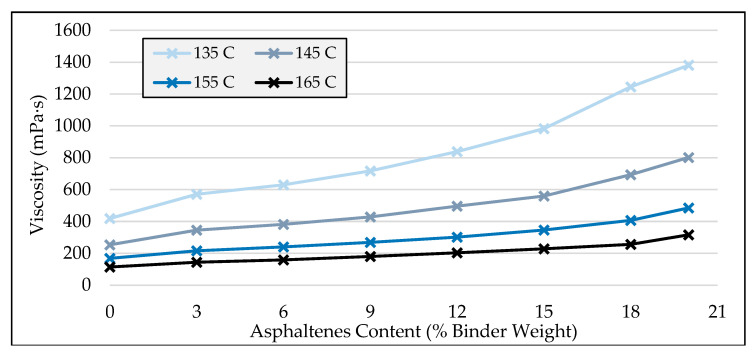
High-temperature viscosity of asphaltenes-modified binders versus asphaltenes content.

**Figure 7:**
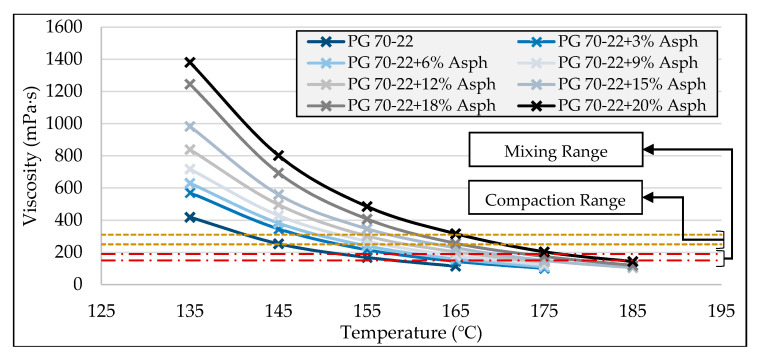
High-temperature viscosity of asphaltenes-modified binders versus temperature.

High-temperature viscosities of modified binders over a wide range of temperatures for different asphaltenes content are plotted and presented in Figure 6 and Figure 7 in relation to binder content and temperature, respectively. From Figure 6, it can be seen that, at all asphaltenes contents, the workability criterion of viscosity being less than 3 Pa·s at 135 °C, as specified in AASHTO M320 standard [45], was satisfied.

As shown in Figure 6 and Figure 7, it was observed that, generally, the addition of asphaltenes increases the viscosity of the asphalt binder. Furthermore, it can be seen that the effect of asphaltenes on viscosity was found to be more evident at higher asphaltenes contents and at lower temperatures. Moreover, the viscosities of different binders were found to exhibit a converging trend as the temperature increased, as seen in Figure 7. This shows that, as the temperature increased, the effect of asphaltenes content on the viscosity of the binder was found to decrease.

The data shown in Figure 7 were also used to estimate the mixing and compaction temperatures of the asphalt binders containing different asphaltenes contents, and the results are presented in Table 3. As shown in the table, it was found that an increase in the viscosity of the asphalt binders due to the addition of asphaltenes leads to an increase in the mixing and compaction temperature for the modified asphalt binders. Accordingly, the addition of every percent of asphaltenes content to the base binder PG 70–22 on average was found to increase the mixing and compaction temperatures by 1.4 °C and 1.3 °C, respectively.

### 3.5. SARA Analysis

In addition to the base binder PG 70–22, a few modified asphalt binders with higher asphaltenes contents were selected for SARA analysis, because of their superior performances at high temperatures. Those selected were the asphalt binders with 12%, 18%, and 20% asphaltenes content. The asphaltenes were also tested for SARA content in order to determine its purity. The results of these analyses are summarized Table 4.

As can be seen in Table 4, the addition of asphaltenes content was found to noticeably increase the polar fraction of the asphalt binder. Comparing the rheological and SARA analysis results, it can be concluded that the increase in polar fraction content causes the stiffness, elasticity, and viscosity of asphalt binder to increase.

The colloidal index (CI) values for the different asphalt binders were also calculated as presented in Table 4. It was observed that the addition of asphaltenes increases the instability of the asphaltenes particles in the maltenes matrix. Among the asphaltenes-modified binders, the one with 20% asphaltenes content tended to be the most unstable, with a CI value greater than 1.2 [18].

## 4. Conclusions

The effects of asphaltenes at different levels of content on the performance of asphalt binder PG 70–22 at high temperatures have been studied. Based on the analysis conducted in this study, the main conclusions drawn are presented as follows:

- The addition of asphaltenes increases the stiffness and elasticity of the asphalt binder, which in turn results in a considerable improvement in resistance against permanent deformation. On average, a 6% increase in asphaltenes content corresponds to a one-interval increase in high PG temperature grade of the asphalt binder.
- The effect of asphaltenes on high-temperature performance parameters, shear modulus, phase angle, and rutting factor is more pronounced as the binder ages.
- As the temperature decreases, the asphaltenes exhibit a stronger impact in terms of increasing the asphalt binder’s stiffness and elasticity due to aging. Since higher values of stiffness and elasticity adversely affect the cracking resistance at lower temperatures, this indicates that the possibility of cracking would increase at lower temperatures with increased asphaltenes content.
- The use of asphaltenes to modify asphalt binder serves to increase the binder’s high-temperature viscosity. The effect of asphaltenes on the binder viscosity is more evident at higher levels of asphaltenes content and lower temperatures.
- Comparing the rheological and SARA analysis results, it can be concluded that the increase in polar fraction content due to the addition of asphaltenes, causes the stiffness, elasticity, and viscosity of the asphalt binder to increase.
- From the CI values, it can be concluded that the addition of asphaltenes increases the instability of the asphaltenes particles in the maltenes matrix; a phenomenon which is largely the result of polarity level differences between the asphaltenes and other fractions of the asphalt binder.

## Figures and Tables

**Table 1:** Specifications of the PG 70–22 binder [29].

Property	ASTM	Specification		Typical Value
		Minimum	Maximum	
Density @ 15 °C, kg/L	D 70 [30]	---	---	1.0341
Penetration @ 25 °C (100 g, 5 s), dmm	D 5 [31]	80	100	90
Flash Point (COC), °C	D 92 [32]	230	---	276
Ductility @ 25 °C (5cm/min), cm	D 113 [33]	100	---	150+
Solubility in trichloroethylene, %	D 2042 [34]	99.5	---	99.9
Absolute viscosity @ 60 °C, Pa.s	D 2171 [35]	150	---	183
Viscosity @ 135 °C, Pa.s	D 4402 [36]	---	3.00	0.42
Mass loss, %	D 1754 [37]	---	1.0	0.37

**Table 2:** High PG grading results for PG 70–22 modified with asphaltenes.

Binder + Asphaltenes, (% Binder wt)	High PG Grade (Unaged Binder) °C	High PG Grade (RTFO-Aged Binder) °C	Continuous High PG Grade °C	Standard High PG Grade °C
0% Asph	70.5	71.1	70.5	70
3% Asph	73.9	75.1	73.9	70
6% Asph	76.5	78.2	76.5	76
9% Asph	81.3	81.1	81.1	76
12% Asph	82.9	84.6	82.9	82
15% Asph	85.9	87.6	85.9	82
18% Asph	88.0	90.3	88.0	88
20% Asph	89.9	92.6	89.9	88

**Table 3:** Mixing and compaction temperature range of base and asphaltenes-modified binders.

Binder + Asphaltenes, (% Binder wt)	Mixing Temperature Range (°C)	Compaction Temperature Range (°C)
0% Asph	152–158	141–145
3% Asph	158–164	147–152
6% Asph	161–167	149–154
9% Asph	164–170	152–157
12% Asph	167–173	154–160
15% Asph	169–175	158–163
18% Asph	173–179	161–166
20% Asph	177–184	165–170

**Table 4:** SARA analysis results for select asphalt binders.

Binder/Material	Saturate	Aromatic	Resin	Asphaltenes	Polar Fraction	Non-Polar Fraction	Colloidal Index
Asphaltenes	6.85	9.68	3.84	79.62	83.46	16.53	6.40
0% Asph	25.41	20.36	31.58	22.59	54.17	45.77	0.92
12% Asph	21.63	20.94	24.75	32.17	56.92	42.57	1.18
18% Asph	18.83	20.25	25.23	35.16	60.39	39.08	1.19
20% Asph	17.93	19.74	21.57	39.76	61.33	37.67	1.40

## References

[1] Kocak S., Kutay M. (2020). Fatigue performance assessment of recycled tire rubber modified asphalt mixtures using viscoelastic continuum damage analysis and AASHTOWare pavement ME design. Constr. Build. Mater..

[2] Liu H., Chen Z., Wang Y., Zhang Z., Hao P. (2018). Effect of poly phosphoric acid (PPA) on creep response of base and polymer modified asphalt binders/mixtures at intermediate-low temperature. Constr. Build. Mater..

[3] Hunter R.N., Self A., Read J. (2015). The Shell Bitumen Handbook.

[4] Junaid M., Irfan M., Ahmed S., Ali Y. (2018). Effect of binder grade on performance parameters of asphaltic concrete paving mixtures. Int. J. Pavement Res. Technol..

[5] Vamegh M., Ameri M., Naeni S.F.C. (2019). Performance evaluation of fatigue resistance of asphalt mixtures modified by SBR/PP polymer blends and SBS. Constr. Build. Mater..

[6] Siddig E.A., Feng C.P., Ming L.Y. (2018). Effects of ethylene vinyl acetate and nanoclay additions on high-temperature performance of asphalt binders. Constr. Build. Mater..

[7] Polacco G., Filippi S., Merusi F., Stastna G. (2015). A review of the fundamentals of polymer-modified asphalts: Asphalt/polymer interactions and principles of compatibility. Adv. Colloid Interface Sci..

[8] Firoozifar S.H., Foroutan S., Foroutan S. (2011). The effect of asphaltene on thermal properties of bitumen. Chem. Eng. Res. Des..

[9] Behnood A., Gharehveran M.M. (2019). Morphology, rheology, and physical properties of polymer-modified asphalt binders. Eur. Polym. J..

[10] Padhan R.K., Sreeram A. (2018). Enhancement of storage stability and rheological properties of polyethylene (PE) modified asphalt using cross linking and reactive polymer based additives. Constr. Build. Mater..

[11] Ghasemirad A., Asgharzadeh S.M., Tabatabaee N. (2017). A comparative evaluation of crumb rubber and devulcanized rubber modified binders. Pet. Sci. Technol..

[12] Choudhary J., Kumar B., Gupta A. (2020). Utilization of solid waste materials as alternative fillers in asphalt mixes: A review. Constr. Build. Mater..

[13] Kalantar Z.N., Karim M.R., Mahrez A. (2012). A review of using waste and virgin polymer in pavement. Constr. Build. Mater..

[14] Meisen A. (2017). Bitumen Beyond Combustion (BBC) Project Phase 1 Report.

[15] Ashtari M. (2016). New pathways for asphaltenes upgrading via oxy-cracking in liquid phase. Chemical Engineering.

[16] Alipour M., Kurian V., Dhir S., Gupta R. (2016). Analysis of syngas cooler fouling from asphaltene gasification. Fuel Process. Technol..

[17] Yang C., Xie J., Wu S., Amirkhanian S., Zhou X., Ye Q., Yang D., Hu R. (2020). Investigation of physicochemical and rheological properties of SARA components separated from bitumen. Constr. Build. Mater..

[18] Lesueur D. (2009). The colloidal structure of bitumen: Consequences on the rheology and on the mechanisms of bitumen modification. Adv. Colloid Interface Sci..

[19] Michalica P., Kazatchkov I.B., Stastna J., Zanzotto L. (2008). Relationship between chemical and rheological properties of two asphalts of different origins. Fuel.

[20] Xu Y., Zhang E., Shan L. (2019). Effect of SARA on Rheological Properties of Asphalt Binders. J. Mater. Civ. Eng..

[21] Sultana S., Bhasin A. (2014). Effect of chemical composition on rheology and mechanical properties of asphalt binder. Constr. Build. Mater..

[22] Ramírez-Corredores M.M. (2017). The Science and Technology of Unconventional Oils: Finding Refining Opportunities.

[23] Airey G. (2004). Styrene butadiene styrene polymer modification of road bitumens. J. Mater. Sci..

[24] Mangiafico S., Di Benedetto H., Sauzéat C., Olard F., Pouget S., Planque L. (2016). Effect of colloidal structure of bituminous binder blends on linear viscoelastic behaviour of mixtures containing reclaimed asphalt pavement. Mater. Des..

[25] Varanda C., Portugal I., Ribeiro J., Silva A., Silva C.M. (2016). Influence of polyphosphoric acid on the consistency and composition of formulated bitumen: Standard characterization and NMR insights. J. Anal. Methods Chem..

[26] Hofko B., Eberhardsteiner L., Füssl J., Grothe H., Handle F., Hospodka M., Grossegger D., Nahar S.N., Schmets A.J.M., Scarpas A. (2016). Impact of maltene and asphaltene fraction on mechanical behavior and microstructure of bitumen. Mater. Struct..

[27] Eberhardsteiner L., Füssl J., Hofko B., Handle F., Hospodka M., Blab R., Grothe H. (2015). Influence of asphaltene content on mechanical bitumen behavior: Experimental investigation and micromechanical modeling. Mater. Struct..

[28] Robertson R.E., Branthaver J.F., Plancher H., Duvall J.J., Ensley E.K., Harnsberger P.M. (1991). Chemical Properties of Asphalts and Their Relationship to Pavement Performance.

[29] (2019). Specifications and Typical Anlyses of an Asphalt Cement 80/100 A PEN used in the Construction of Pavements.

[30] ASTM D70-18 (2018). Standard Test Method for Density of Semi-Solid Asphalt Binder (Pycnometer Method).

[31] ASTM D5-05 (2005). Standard Test Method for Penetration of Bituminous Materials.

[32] ASTM D92-18 (2018). Standard Test Method for Flash and Fire Points by Cleveland Open Cup Tester.

[33] ASTM D113-17 (2017). Standard Test Method for Ductility of Asphalt Materials.

[34] ASTM D2042-15 (2015). Standard Test Method for Solubility of Asphalt Materials in Trichloroethylene.

[35] ASTM D2171-18 (2018). Standard Test Method for Viscosity of Asphalts by Vacuum Capillary Viscometer.

[36] ASTM D4402-15 (2015). Standard Test Method for Viscosity Determination of Asphalt at Elevated Temperatures Using a Rotational Viscometer.

[37] ASTM D1754-14 (2014). Standard Test Method for Effects of Heat and Air on Asphaltic Materials (Thin-Film Oven Test).

[38] Zuo P., Qu S., Shen W. (2019). Asphaltenes: Separations, structural analysis and applications. J. Energy Chem..

[39] Speight J.G. (2004). Petroleum Asphaltenes-Part 1: Asphaltenes, resins and the structure of petroleum. Oil Gas Sci. Technol..

[40] AASHTO T240-13 (2017). Effect of Heat and Air on a Moving Film of Asphalt Binder (Rolling Thin-Film Oven Test). AASHTO Provisional Standards.

[41] Zhang H., Chen Z., Xu G., Shi C. (2018). Physical, rheological and chemical characterization of aging behaviors of thermochromic asphalt binder. Fuel.

[42] Zhao X., Wang S., Wang Q., Yao H. (2016). Rheological and structural evolution of SBS modified asphalts under natural weathering. Fuel.

[43] Yu J.Y., Feng P.C., Zhang H.L., Wu S.P. (2009). Effect of organo-montmorillonite on aging properties of asphalt. Constr. Build. Mater..

[44] AASHTO T315-12 (2016). Determining the Rheological Properties of Asphalt Binder Using a Dynamic Shear Rheometer (DSR). AASHTO Provisional Standards.

[45] AASHTO M320-17 (2017). Performance-Graded Asphalt Binder. AASHTO Provisional Standards.

[46] AASHTO T316-13 (2017). Viscosity Determination of Asphalt Binder Using Rotational Viscometer. AASHTO Provisional Standards.

[47] ASTM D6560-17 (2017). Standard Test Method for Determination of Asphaltenes (Heptane Insolubles) in Crude Petroleum and Petroleum Products.

[48] Wang J., Wang T., Hou X., Xiao F. (2019). Modelling of rheological and chemical properties of asphalt binder considering SARA fraction. Fuel.

[49] Siddiqui M.N., Ali M.F. (1999). Studies on the aging behavior of the Arabian asphalts. Fuel.

